# Prognostic value of normal levels of preoperative tumor markers in colorectal cancer

**DOI:** 10.1038/s41598-023-49832-5

**Published:** 2023-12-20

**Authors:** Guangming Ren, Ruikai Li, Gaozan Zheng, Kunli Du, Hanjun Dan, Hongze Wu, Xinyu Dou, Lili Duan, Zhenyu Xie, Liaoran Niu, Ye Tian, Jianyong Zheng, Fan Feng

**Affiliations:** 1 Department of Gastrointestinal Surgery, Xijing Hospital, Fourth Military Medical University, Xi’an, China; 2 Xi’an Medical University, Xi’an, China

**Keywords:** Prognostic markers, Colorectal cancer, Cancer models, Tumour biomarkers, Colorectal cancer, Prognostic markers

## Abstract

Carcinoembryonic antigen (CEA), carbohydrate antigen 19-9 (CA19-9), carbohydrate antigen 125 (CA125), and alpha-fetoprotein (AFP) are widely used tumor markers for colorectal cancer (CRC), but their clinical significance is unknown when the levels of these tumor markers were within the normal range. This retrospective study included 2145 CRC patients. The entire cohort was randomly divided into training and validation datasets. The optimal cut-off values of tumor markers were calculated using X-tile software, and univariate and multivariate analyses were performed to assess its association with overall survival (OS). The nomogram model was constructed and validated. The entire cohort was randomly divided into a training dataset (1502 cases, 70%) and a validation dataset (643 cases,30%). Calculated from the training dataset, the optimal cut-off value was 2.9 ng/mL for CEA, 10.1 ng/mL for CA19-9, 13.4 U/mL for CA125, and 1.8 ng/mL for AFP, respectively. Multivariate analysis revealed that age, tumor location, T stage, N stage, preoperative CA19-9, and CA125 levels were independent prognostic predictors. Even within the normal range, CRC patients with relatively high levels of CA19-9 or CA125 worse OS compared to those with relatively low levels. Then, based on the independent prognostic predictors from multivariate analysis, two models with/without (model I/II) CA19-9 and CA125 were built, model I showed better prediction and reliability than model II. Within the normal range, relatively high levels of preoperative CA19-9 and CA125 were significantly associated with poor OS in CRC patients. The nomogram based on CA19-9 and CA125 levels showed improved predictive accuracy ability for CRC.

## Introduction

Colorectal cancer (CRC) is currently the third leading cause of cancer globally, and it represents the second highest number of cancer-related deaths worldwide. In recent years, the incidence and mortality rates of CRC are steadily increasing, constituting a substantial menace to public health^1^. Owing to the absence of distinct symptoms during the initial phases of CRC, the rate of diagnosis remains depressingly low, with most patients presenting themselves for medical evaluation only when the disease has already progressed to advanced stages. Consequently, this delay results in the missed opportunity for optimal therapeutic interventions. Despite combinations of many advanced therapies for CRC have improved early detection and treatment, radical surgery is still the optimal treatment and the prognosis of patients is still not promising^2^. Moreover, accurate prediction of the prognosis is of great importance for individualized treatment for CRC^3^.

Tumor markers can indicate the presence of cancer, more importantly, provide information about treatment response or progression^4^. Carcinoembryonic antigen (CEA)^5^, carbohydrate antigen 19-9 (CA19-9)^6^, carbohydrate antigen 125 (CA125)^7^, and alpha-fetoprotein (AFP)^8^ can be elevated in CRC patients, making them widely employed in the diagnosis and monitoring of CRC. Previous studies have shown that elevated levels of preoperative CEA, CA19-9, CA125, and AFP were associated with worse prognosis of CRC patients^7,9–12^. However, the prognostic value of preoperative CEA, CA19-9, CA125, and AFP with the normal range for CRC patients is unknown. Up to date, only two studies investigated the association between normal levels of CEA and the survivals of CRC patients^13,14^ and found that the prognosis of patients with relatively high levels of preoperative CEA was worse than that with relatively low levels of CEA in CRC patients. Therefore, the purpose of this study was to investigate the association between normal levels of preoperative serum CEA, CA19-9, CA125, and AFP and the prognosis of CRC patients after radical resection.

## Patients and methods

**Figure 1:**
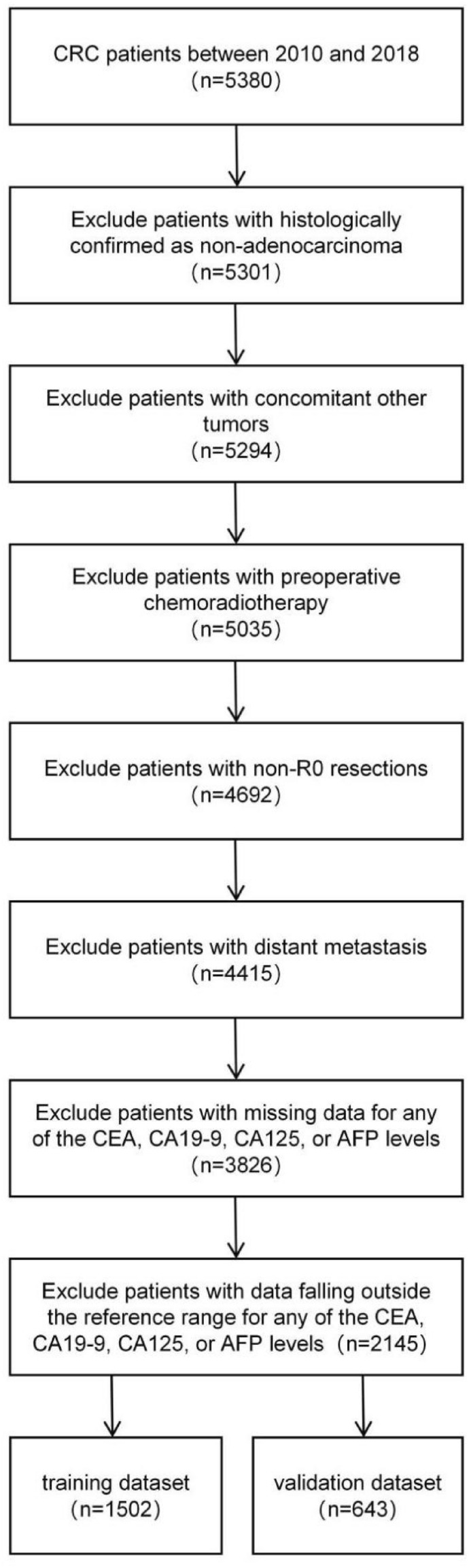
Flowchart of the CRC patients with training and validation datasets. CRC, Colorectal cancer; CEA, carcinoembryonic antigen; CA19-9, carbohydrate antigen 19-9; CA125, carbohydrate antigen 125; AFP, alpha-fetoprotein.

From January 2010 to December 2018, a total of 5380 CRC patients treated with radical resection in the Department of Digestive Surgery, Xijing Hospital. Based on the inclusion criteria, a final selection yielded 2145 patients included in this study. The inclusion criteria were listed as follows: (1) histologically confirmed as colorectal adenocarcinoma, (2) without distant metastasis, (3) without other tumors, (4) without preoperative chemoradiotherapy, (5) with R0 resection, (6) levels of preoperative CEA, CA19-9, CA125 and AFP were all within the reference range, (7) with complete follow-up data. And Fig. 1 presents the flowchart of the selection process. The study was approved by the Ethics Committee of Xijing Hospital (ethical code: KY20222260-C-1). Informed consent was obtained from patients before operation.

Clinicopathological data, including age, gender, tumor size, operative method, tumor location, differentiation status, T stage, N stage, TNM stage and levels of CEA, CA19-9, CA125 and AFP were recorded. The concentrations of CEA, CA19-9, CA125, and AFP were obtained using the Electrochemiluminescence Immunoassay (ECLIA) method. The reference range for tumor markers, considered as the normal range, is as follows: CEA ≤ 5 ng/mL, CA19-9 ≤ 27 U/mL, CA125 ≤ 35 U/mL, AFP ≤ 8.1 ng/mL, and all these markers were measured in Xijing hospital within 3 weeks before surgery. The patients were followed up every 3 months in the first 3 years and every 6 months thereafter. Overall survival (OS) is the time between the date of surgery and death from any cause.

The dataset was randomly divided into a training dataset (70%, n = 1502) and a validation dataset (30%, n = 643) using the “rand” function in Microsoft Excel. Differences in the distribution of variables between the training and validation dataset were analyzed by SPSS software (version 26, SPSS Inc. USA) using chi-square tests. The optimal cut-off values of levels of CEA, CA19-9, CA125 and AFP were calculated using X-Tile software (Yale University, V3.6.1) based on the training dataset. Risk factors with a *p*-value <0.1 in univariate analysis were included in the Cox model multivariate analysis. Factors with a p-value < 0.05 were considered as independent risk factor in the multivariate analysis. Nomogram was constructed by R software (www.r-project.org, version 4.0.5) based on independent risk factors. The predictive accuracy of nomograms was measured by calibration curve and concordance index (C-index) in the training and validation dataset. The area under curve (AUC) of the receiver operating characteristic (ROC) curve was calculated to compare the predictive accuracy between the two models. OS was assessed by the Kaplan-Meier method through GraphPad Prism 8 (GraphPad Software, Inc., USA). A *p*-value < 0.05 was considered statistically significant.

### Ethical approval and consent to participate, informed consent

The chart review did not personally identifiable data, and its findings were presented as averaged datasets for each center. The study was conducted in accordance with the Declaration of Helsinki, and approved by the Ethics Committee of Xijing Hospital (ethical code: KY20222260-C-1,2022.11.07). Informed consent was obtained from all individual participants included in the study. Informed consent was obtained from all subjects involved in the study.

## Results

**Figure 2:**
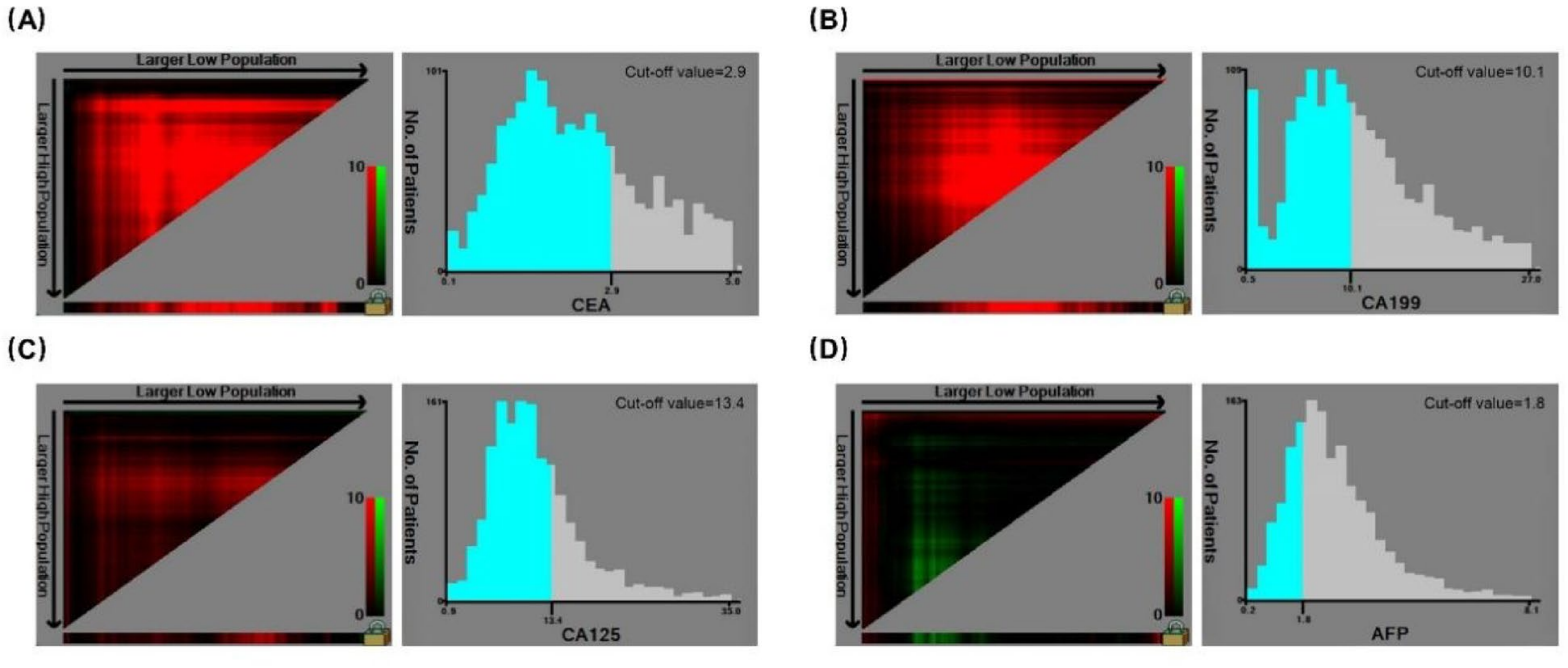
Cut-off value of serum CEA and CA19-9, CA125, and AFP levels for the OS of CRC as calculated using X-tile. CRC, Colorectal cancer; CEA, carcinoembryonic antigen; CA19-9, carbohydrate antigen 19-9; CA125, carbohydrate antigen 125; AFP, alpha-fetoprotein; OS, overall survival.

There were 1269 male (59.2%) and 876 (40.8%) female. The median age was 61 years (range 21–93 years). The follow-up period ranged from 2.6 to 144.6 months with a mean of 65.7 months and a median of 64.8 months. During follow-up, 390 patients died, accounting for 18.2% of the entire cohort. The 5-, and 10-year OS rate was 84.6% and 74.4%, respectively. The study cohort was randomly divided into a training dataset (1502 cases, 70%) and a validation dataset (643 cases, 30%). Calculated from the training dataset, the optimal cut-off value was 2.9 ng/mL for CEA, 10.1 ng/mL for CA19-9, 13.4 U/mL for CA125 and 1.8ng/mL for AFP, respectively (Fig 2). All the parameters were comparable between the two datasets (Table 1).

**Table 1:** Clinicopathological characteristics of the entire cohort and the comparison of variable consistency between training and validation datasets.

Variables	Total (%)	Training (%)	Validation (%)	*P*-value
	N = 2145	N = 1502	N = 643	
Age (years)				0.713
< 70	1639 (76.4)	1151 (76.6)	488 (75.9)	
≥ 70	506 (23.6)	351 (23.4)	155 (24.1)	
Gender				0.893
Male	1269 (59.2)	890 (59.3)	379 (58.9)	
Female	876 (40.8)	612 (40.7)	264 (41.1)	
Tumor size (cm)				0.668
< 5	1515 (70.6)	1065 (70.9)	450 (70.0)	
≥ 5	630 (29.4)	437 (29.1)	193 (30.0)	
Operative method				0.155
Open	746 (34.8)	508 (33.8)	238 (37.0)	
Laparoscopic	1399 (65.2)	994 (66.2)	405 (63.0)	
Tumor location				0.359
Right-sided colon	412 (19.2)	281 (18.7)	131 (20.4)	
Left-sided colon	434 (20.2)	315 (21.0)	119 (18.5)	
Rectum	1299 (60.6)	906 (60.3)	393 (61.1)	
Differentiation status				0.619
Well	253 (11.8)	180 (12.0)	73 (11.4)	
Moderate	1506 (70.2)	1062 (70.7)	444 (69.1)	
Poor	200 (9.3)	133 (8.9)	67 (10.4)	
Mucinous	186 (8.7)	127 (8.5)	59 (9.2)	
T stage				0.375
Tis/T1	252 (11.7)	178 (11.9)	74 (11.5)	
T2	505 (23.5)	353 (23.5)	152 (23.6)	
T3	1239 (57.8)	876 (58.3)	363 (56.5)	
T4	149 (6.9)	95 (6.3)	54 (8.4)	
N stage				0.935
N0	1396 (65.1)	975 (64.9)	421 (65.5)	
N1	529 (24.7)	372 (24.8)	157 (24.4)	
N2	220 (10.3)	155 (10.3)	65 (10.1)	
Stage (AJCC 8th edition)				0.991
I	621 (29.0)	436 (29.0)	185 (28.8)	
II	775 (36.1)	539 (35.9)	236 (36.7)	
III	749 (34.9)	527 (35.1)	222 (34.5)	
Preoperative CEA				0.816
< 2.9 ng/mL	1522(71.0)	1068 (71.1)	454 (70.6)	
≥ 2.9 ng/mL	623(29.0)	434 (28.9)	189 (29.4)	
Preoperative AFP				0.167
< 1.8 ng/mL	613 (28.6)	416 (27.7)	197 (30.6)	
≥ 1.8 ng/mL	1532 (71.4)	1086 (72.3)	446 (69.4)	
Preoperative CA125				0.713
< 13.4 U/mL	1523 (71.0)	1070 (71.2)	453 (70.5)	
≥ 13.4 U/mL	622 (29.0)	432 (28.8)	190 (29.5)	
Preoperative CA19-9				0.696
< 10.1 U/mL	1158 (54.0)	815 (54.3)	343 (53.3)	
≥ 10.1 U/mL	987 (46.0)	687 (45.7)	300 (46.7)	

AJCC, American Joint Committee on Cancer; CEA, carcinoembryonic antigen; CA19-9, carbohydrate antigen 19–9; CA125, carbohydrate antigen 125; AFP, alpha-fetoprotein.

Univariate analysis revealed that age, tumor location, differentiation status, T stage, N stage, preoperative CEA, CA19-9 and CA125 levels were associated with the prognosis of patients (*p*-value < 0.1) (Table 2). Multivariate analysis showed that age, tumor location, T stage, N stage, preoperative CA19-9 and CA125 levels were independent prognostic predictors (*p*-value < 0.05) (Table 3).

**Table 2:** Univariate analyses of OS in the training datasets.

Variables	β	HR (95%CI)	*P*-value
Age (years)			
< 70		Reference	
≥ 70	0.582	1.789 (1.382–2.318)	< 0.001
Gender			
Male		Reference	
Female	− 0.027	0.973 (0.757–1.251)	0.832
Tumor size (cm)			
< 5		Reference	
≥ 5	–0.198	0.821 (0.619–1.088)	0.170
Operative method			
Open		Reference	
Laparoscopic	− 0.014	0.986 (0.762–1.275)	0.914
Tumor location			0.010
Right-sided colon		Reference	
Left-sided colon	0.377	1.459 (0.930–2.287)	0.100
Rectum	0.579	1.784 (1.217–2.615)	0.003
Differentiation status			0.004
Well		Reference	
Moderate	− 0.018	0.982 (0.672–1.436)	0.927
Poor	0.649	1.914 (1.184–3.093)	0.008
Mucinous	0.155	1.168 (0.683–1.996)	0.570
T stage			< 0.001
Tis/T1		Reference	
T2	0.420	1.523 (0.811–2.858)	0.191
T3	1.073	2.924 (1.662–5.142)	< 0.001
T4	1.847	6.338 (3.360–11.955)	< 0.001
N stage			< 0.001
N0		Reference	
N1	0.842	2.321 (1.734–3.106)	< 0.001
N2	1.684	5.384 (3.967–7.308)	< 0.001
Preoperative CEA			
< 2.9 ng/mL		Reference	
≥ 2.9 ng/mL	0.459	1.582 (1.227–2.041)	< 0.001
Preoperative AFP			
< 1.8 ng/mL		Reference	
≥ 1.8 ng/mL	− 0.214	0.807 (0.622–1.048)	0.107
Preoperative CA125			
< 13.4 U/mL		Reference	
≥ 13.4 U/mL	0.346	1.414 (1.093–1.829)	0.008
Preoperative CA19-9			
< 10.1 U/mL		Reference	
≥ 10.1 U/mL	0.550	1.733 (1.351–2.223)	< 0.001

β, Beta; HR, Hazard Ratio; CI, Confidence Interval; CEA, carcinoembryonic antigen; CA19-9, carbohydrate antigen 19–9; CA125, carbohydrate antigen 125; AFP, alpha-fetoprotein.

**Table 3:** Multivariate analyses of overall survival (OS) in the training datasets.

Variables	β	HR (95%CI)	*P*-value
Age (years)			
< 70		Reference	
≥ 70	0.609	1.838 (1.440–2.397)	< 0.001
Tumor location			< 0.001
Right-sided colon		Reference	
Left-sided colon	0.249	1.282 (0.810–2.031)	0.298
Rectum	0.730	2.074 (1.378–3.122)	< 0.001
Differentiation status			0.287
Well		Reference	
Moderate	-0.196	0.822 (0.555–1.218)	0.329
Poor	0.157	1.170 (0.706–1.939)	0.541
Mucinous	-0.094	0.911 (0.519–1.598)	0.744
T stage			< 0.001
Tis/T1		Reference	
T2	0.286	1.331 (0.699–2.532)	0.384
T3	0.847	2.334 (1.289–4.224)	0.005
T4	1.381	3.979 (1.997–7.930)	< 0.001
N stage			< 0.001
N0		Reference	
N1	0.601	1.825 (1.344–2.477)	< 0.001
N2	1.248	3.484 (2.471–4.913)	< 0.001
Preoperative CEA			
< 2.9 ng/mL		Reference	
≥ 2.9 ng/mL	0.191	1.210 (0.932–1.572)	0.152
Preoperative CA125			
< 13.4 U/mL		Reference	
≥ 13.4 U/mL	0.363	1.437 (1.103–1.872)	0.007
Preoperative CA19-9			
< 10.1 U/mL		Reference	
≥ 10.1 U/mL	0.396	1.486 (1.153–1.915)	0.002

β, Beta; HR, Hazard Ratio; CI, Confidence Interval; CEA, carcinoembryonic antigen; CA19-9, carbohydrate antigen 19-9; CA125, carbohydrate antigen 125.

**Figure 3:**
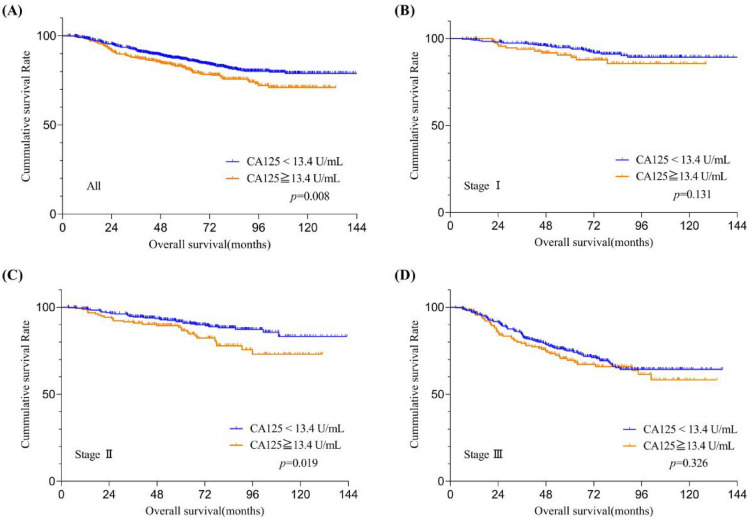
Overall survival curves stratifified by preoperative serum CA125 levels according to tumor stage. Patients with (**A**) all training datasets and American Joint Committee on Cancer 7th stage (**B**) I, (**C**) II, and (**D**) III in the training datasets. CA125,carbohydrate antigen 125.

**Figure 4:**
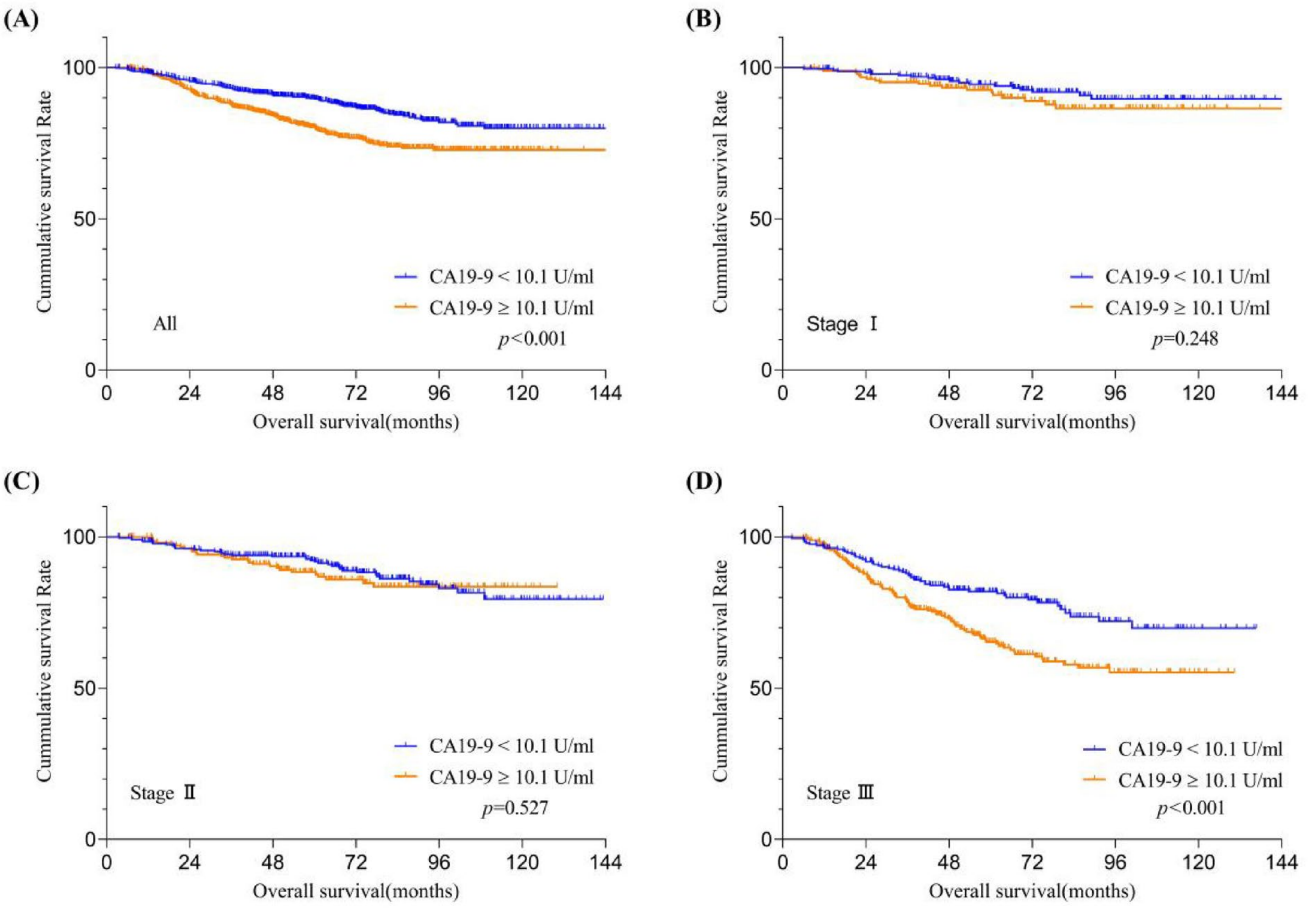
Overall survival curves stratifified by preoperative serum CA19-9 levels according to tumor stage. Patients with (**A**) all training datasets and American Joint Committee on Cancer 7th stage (**B**) I, (**C**) II, and (**D**) III in the training datasets. CA19-9, carbohydrate antigen 19-9.

The OS of patients stratified by CA19-9 and CA125 levels were shown in Figs. 3A and 4A. The OS of patients with relatively high CA19-9 or CA125 levels was significantly worse than that with relatively low levels (both *p* < 0.05). Subgroup analysis based on the TNM stage system reveals that among stage II CRC patients, those with relatively high preoperative serum CA125 levels exhibit a significantly lower OS rate compared to patients that with relatively low levels (Fig. 3C, *p* = 0.019); however, this phenomenon was not observed in stage I (Fig. 3B, *p* = 0.131) and stage III (Fig. 3D, *p* = 0.326) CRC patients. Similarly, in stage III CRC patients, those with relatively high preoperative serum CA19-9 levels exhibit a significantly lower OS rate compared to patients that with relatively low levels (Fig. 4D, *p* < 0.001); yet, this trend was not discerned in stage I (Fig. 4B, *p* = 0.248) and stage II (Fig. 4C, *p* = 0.527) CRC patients.

**Figure 5:**
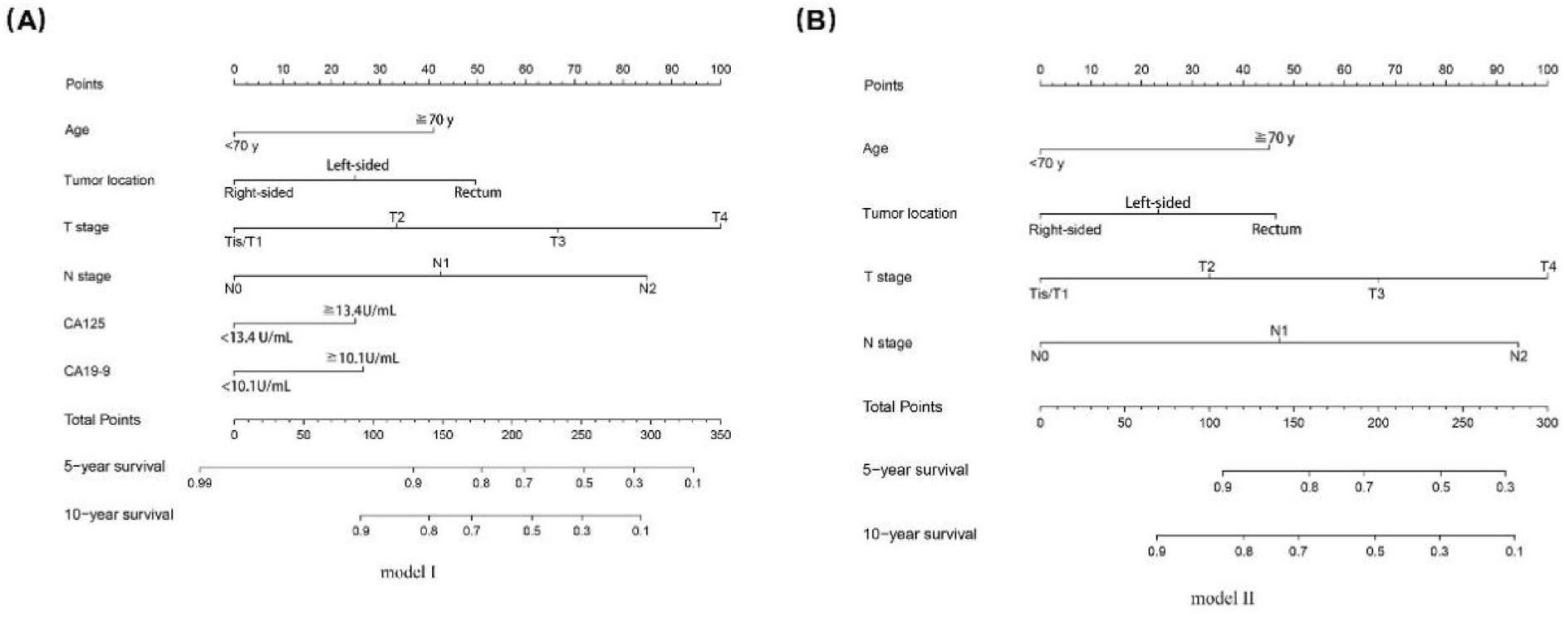
Two nomograms for predicting the 5- and 10-year OS of the training dataset. CA19-9, carbohydrate antigen 19-9; CA125, carbohydrate antigen 125.

**Figure 6:**
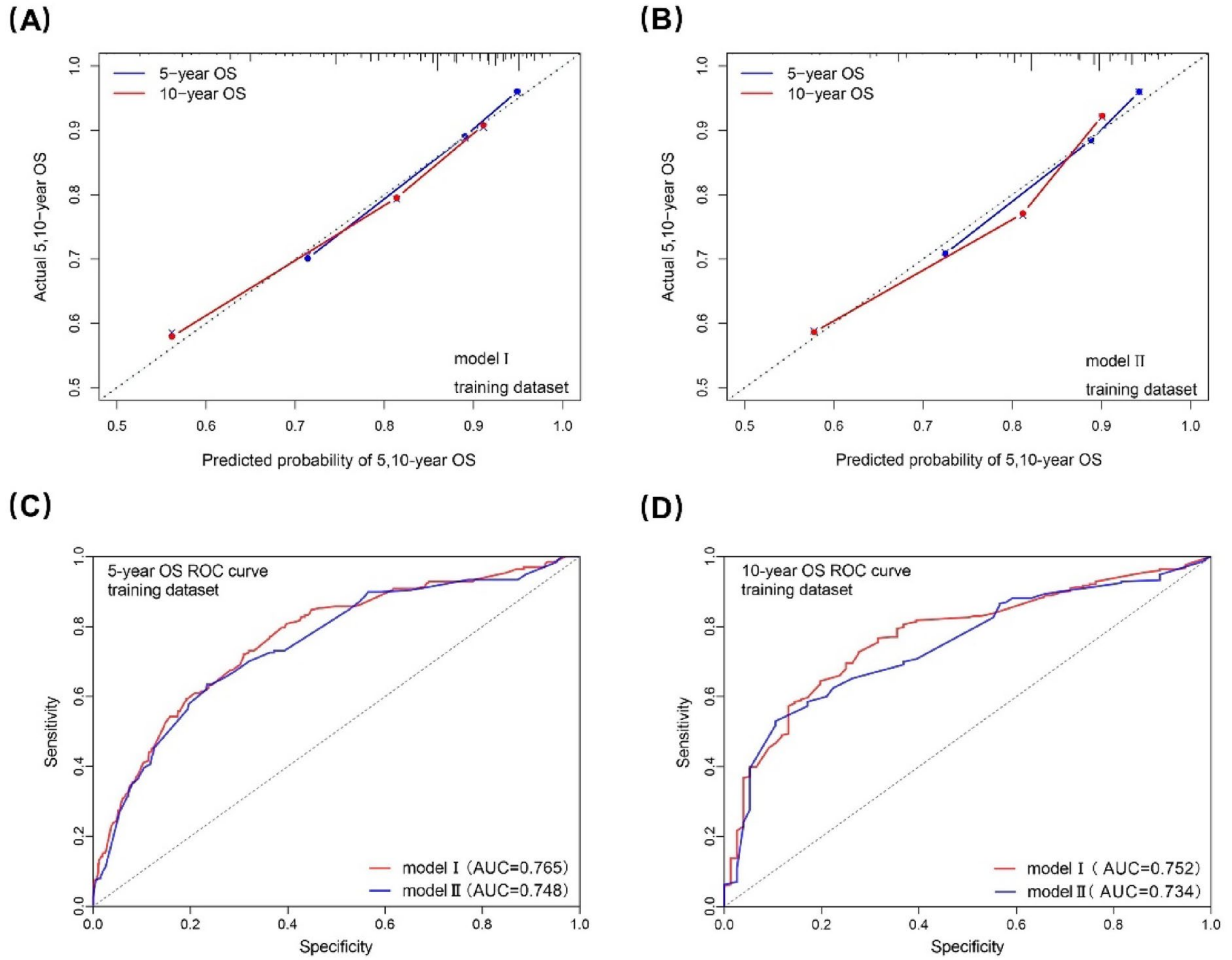
Calibration curves and ROC curves to model I/II for 5-, and 10-year OS in the training dataset. ROC, receiver operating characteristic; OS, overall survival; AUC, area under curve.

Two nomogram models with/without (model I/II) CA19-9 and CA125 were built based on the independent prognostic predictors from multivariate analysis (Fig. 5). The performance of model I/II were assessed with C-index, calibration curve and AUC. In the training dataset, the C-index was 0.734(0.701-0.766) and 0.729(0.695-0.763) for model I and II, which indicated that both the two models had good predictive discrimination. Furthermore, the calibration curve showed a high consistency between prediction and actual observation in both of the two models (Fig. 6A,B). According to ROC curve analysis, AUC about the 5- and 10-year OS prediction of model I was higher than that of model II (0.765 vs 0.747 and 0.773 vs 0.746, respectively), which indicated that model I was performed better than model II in the training dataset (Fig. 6C,D).

**Figure 7:**
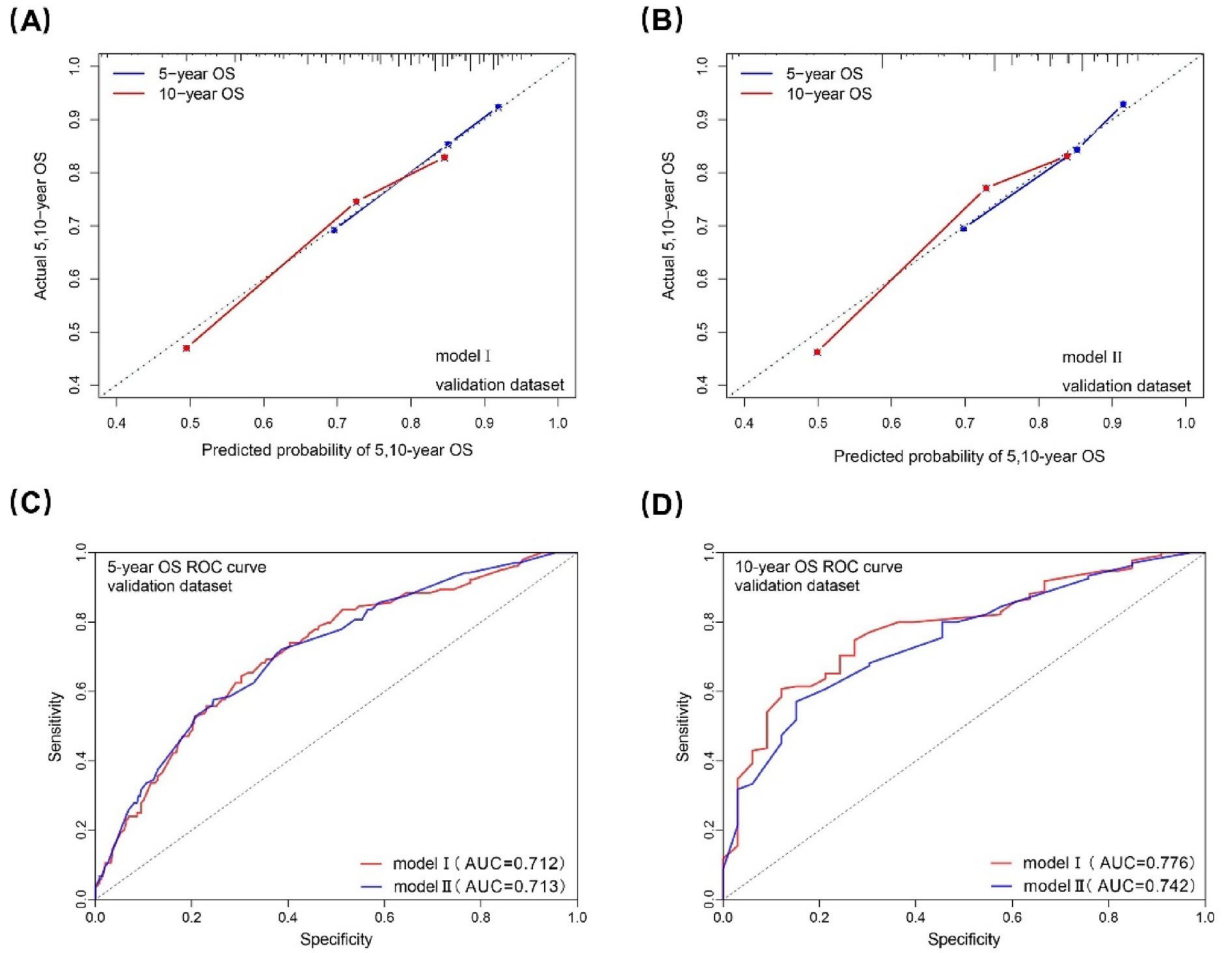
Calibration curves and ROC curves to model I/II for 5-, and 10-year OS in the validation dataset. ROC, receiver operating characteristic; OS, overall survival; AUC, area under curve.

In the validation dataset, the C-index for model I and II was 0.691(0.644-0.739) and 0.699(0.650-0.748). The calibration curve also showed a high consistency between prediction and actual observation in both the two models (Fig. 7A,B). According to the ROC curve analysis, the AUC for the 5-year OS prediction of model I was comparable to that of model II (0.712 vs 0.713), and the AUC for the 10-year OS prediction of model I was higher than that of model II (0.776 vs 0.742), which indicated that model I was still performed better than model II in the validation dataset (Fig. 7C,D).

## Discussion

CEA, CA19-9, CA125 and AFP are cell-surface glycoproteins produced by cancer cells and contributes to the malignant characteristics of tumors^15–17^. As commonly used serum tumor markers, they are easy to detect and convenient to use in clinical work. They are important for treatment planning because they are closely associated with the prognosis of CRC patients^7,10,11^. The association between elevated CEA, CA19-9, CA125 and AFP levels and the prognosis of CRC patients have been explored in a series of studies^18–20^. Only one study examined the prognostic value of preoperative CEA, CA19-9, CA125 and AFP in the normal range for patients with gastric cancer^21^. However, the prognostic value of these tumor markers within the normal range for CRC patients was unclear. The present study explored the association between normal levels of preoperative CEA, CA19-9, CA125 and AFP and prognosis of CRC patients. We found that relatively high levels of preoperative CA19-9 and CA125 were independent risk factors for OS of CRC patients. In addition, a nomogram based on normal CA19-9 and CA125 levels was built and showed improved predictive accuracy and prognostic discriminatory ability for CRC patients.

Serum CEA is the most common biomarker in CRC, and elevated CEA level is indicative of poor prognosis^12^. In fact, the preoperative serum CEA levels were within the normal range in approximately 60%-65% of CRC patients^14,20^. That means the preoperative serum CEA levels could not be used to aid the evaluation of the prognosis of majority of CRC patients. However, two studies investigated the association between the normal preoperative CEA levels and the prognosis of CRC patients recently. One showed that relatively high levels of preoperative serum CEA (2.1 ~ 5 ng/mL) was significantly associated with poor DFS and OS in CRC patients^13^. The other one also found that relatively high levels of preoperative CEA (2.4 ~ 5 ng/mL) was a significant risk factor for OS of the CRC patients^14^. However, the levels of AFP, CA19-9 and CA125 were unclear in the two studies. In our present study, patients with normal CEA levels but with elevated AFP, CA19-9 or CA125 levels were excluded, and the maximum follow-up time after surgery has been increased to 12 years, this may partially explain the different findings about the prognostic value of normal CEA levels between the previous and our present studies.

It is well known that no matter whether the preoperative tumor markers were within the normal range or not, the elevated postoperative tumor markers portend a poor prognosis in CRC^22,23^. However, the clinical significance of postoperative tumor markers within the normal range is unknown.

Univariate and multivariate analyses indicate that preoperative serum levels of CA125 and CA19-9 within the normal range remain significant independent prognostic factors for patients with CRC. This suggests that irrespective of the tumor stage, the overall levels of preoperative serum CA125 and CA19-9 are critical indicators of cancer prognosis. Subgroup analysis based on TNM stage demonstrates a significant relationship between preoperative serum CA125 levels and the prognosis of stage II patients. However, this relationship is not significantly evident in stage I and stage III patients. This could be attributed to the presence of other biological factors that may more profoundly influence the prognosis during the early and later stages of the tumor. Conversely, there is a very strong association between preoperative serum CA19-9 levels and the prognosis of CRC patients, indicating that for patients at a more advanced stage of CRC, CA19-9 might serve as a key biological marker. It can be utilized for prognostic evaluation and potentially constitutes an important consideration for targeted therapy. CA125 and CA19-9 exhibit varying degrees of significance across different tumor stages. When it comes to the treatment and prognostic evaluation of individual patients, it is imperative to make comprehensive judgments on the clinical relevance of these markers, taking into account the specific stage of the tumor and additional clinical information. These disparities also potentially point towards the necessity for further research to comprehensively understand the mechanisms of action and clinical utility values of various biomarkers at different stages.

Due to removal of the tumor, elevated preoperative tumor markers will decline, and the falling of the tumor markers was significantly associated with the prognosis of patients^24^. However, for patients with preoperative tumor markers within the normal range, it remains unclear whether the tumor markers will decline further after resection of tumor. Furthermore, it is also unclear whether there is a relationship between the extent of decline and prognosis of patients. Further studies are needed to investigate these questions.

Featured by visual and mathematical advantages, nomogram facilitates the clinical implementation and probability calculation of risk factor or other predictor variables. Although several nomograms had been developed to predict the OS for peritoneal metastasis, liver metastasis, and stage IV CRC, etc.^25–27^, nomogram for predicting the OS of CRC patients with normal levels of preoperative CEA, CA19-9, CA125 and AFP was lacking. In our present study, the nomogram showed good discriminatory capability and prediction accuracy. The variables in the nomogram can be easily obtained from routine clinical practice without extra financial burden on the patients. As a result, clinicians can use it to make a quick assessment of patient’s prognosis. The nomogram we have developed allows for the rapid calculation of a patient's survival time and probability based on several clinical input data. This tool aids physicians in explaining potential disease progression and prognosis to patients. It not only facilitates communication between doctors and patients but also enables patients to better comprehend their health status. Consequently, patients can participate more actively in the decision-making process regarding treatment options, potentially even reducing the waste of medical resources.

Our study posits that preoperative serum levels of CA19-9 and CA125 within the normal range are instrumental in identifying cohorts of high-risk patients who may require closer monitoring or more aggressive treatment approaches. In cases where CA19-9 and CA125 levels are comparatively elevated, we advocate for more frequent follow-up examinations and dynamic assessments. Moreover, we recognize that decision-making based solely on biomarker levels is insufficient. Instead, this should be a multifaceted decision-making process that comprehensively considers various clinical parameters, the patient's overall health status, and individual differences.

This study has several limitations. First, it was a single-center’s experience which may result in selection bias, so multicenter large-scale studies are needed to verify these findings. Second, the sample size was not large enough, especially for the patients with relatively high levels of CEA or CA125, or relatively low levels of AFP, which may also result in some extent of bias during analysis. Third, some risk factors reported in previous studies which was associated with the prognosis of CRC patients, such as perineural invasion^28,29^, microsatellite stability status^30^ and gene mutational status^31^ were not included in this study because of lack of data.

In conclusion, our study showed that, even within the normal range, relatively high levels of preoperative CA19-9 and CA125 were significantly associated with poor OS of CRC patients. The nomogram which based on CA19-9 and CA125 levels showed improved predictive accuracy and prognostic discriminatory ability for CRC. The findings may provide important indications for clinicians in the prognostic evaluation of CRC patients with normal levels of tumor markers.

## Data Availability

All data described in the manuscript will be freely available to any researcher wishing to use them for non-commercial purposes, without breaching participant confidentiality, and the data can be obtained by contacting the correspondence author.
